# A real-world study for timely assessing the diabetic macular edema refractory to intravitreal anti-VEGF treatment

**DOI:** 10.3389/fendo.2023.1108097

**Published:** 2023-05-17

**Authors:** Tsung-Cheng Hsieh, Guang-Hong Deng, Yung-Ching Chang, Fang-Ling Chang, Ming-Shan He

**Affiliations:** ^1^ Institute of Medical Sciences, Tzu Chi University, Hualien, Taiwan; ^2^ Tzu Chi University Research Center for Big Data Teaching, Research and Statistic Consultation, Hualien, Taiwan; ^3^ Department of Ophthalmology, Buddhist Tzu Chi General Hospital, Hualien, Taiwan; ^4^ Department of Ophthalmology and Visual Science, Tzu Chi University, Hualien, Taiwan

**Keywords:** diabetic macular edema, anti-vegf, optical coherence tomography, epiretinal membrane, diabetic retinopathy

## Abstract

**Background:**

Early Identifying and characterizing patients with diabetic macular edema (DME) is essential for individualized treatment and outcome optimization. This study aimed to timely investigate optical coherence tomography (OCT) biomarkers of DME refractory to intravitreal anti-vascular endothelial growth factor (VEGF) therapy.

**Methods:**

We retrospective reviewed 72 eyes from 44 treatment-naïve patients who were treated with intravitreal anti-VEGF for DME. OCT scans prior to anti-VEGF were evaluated for serous retinal detachment (SRD), size of outer nuclear layer cystoid changes, diffuse retinal thickening, integrity of the inner segment-outer segment (IS-OS) junction, quantity and location of hyperreflective foci, vitreomacular interface abnormalities, and epiretinal membrane (ERM). The Baseline best-corrected visual acuity (BCVA) and central macular thickness was recorded at baseline and 4 months after treatment with anti-VEGF. The main outcome measure was the correlation between spectral-domain OCT measurements and BCVA response at baseline and after anti-VEGF treatment (mean change from baseline; ≥ 10 Early Treatment Diabetic Retinopathy Study letters in BCVA).

**Results:**

Partially continuous IS-OS layers (partially vs. completely continuous: β, -0.138; Wald chi-square, 16.392; P<0.001) was predictor of better response to anti-VEGF treatment. In contrast, ERM (present vs. absent ERM: β, 0.215; Wald chi-square, 5.921; P=0.015) and vitreomacular traction (vitreomacular traction vs. posterior vitreous detachment: β=0.259; Wald chi-square=5.938; P=0.015) were the predictors of poor response. The improvement of BCVA trended toward the OCT predictive value of central macular thickness reduction; however, this was not significant.

**Conclusion:**

Partially continuous IS-OS layers is predictive of better response to anti-VEGF therapy in DME. Meanwhile, ERM is a significant predictor of poor response.

## Introduction

Vision loss associated with diabetic retinopathy (DR) is most commonly caused by diabetic macular edema (DME) (1). The Diabetes Control and Complications Trial (DCCT) reported that 27% of patients with type 1 diabetes developed macular edema within 9 years of diabetes onset (2). Other studies indicate that in type 2 diabetes patients, the prevalence increases from 3% within 5 years of diagnosis to 28% after 20 years (3). Although several treatment options are available, no consensus on DME treatment based on patient status has been achieved.

Vascular endothelial growth factor (VEGF) is an important mediator of abnormal vascular permeability in eyes with DME (4). Anti-VEGF injections are generally proposed as first-line therapy for center-involved DME and are effective in improving visual acuity (VA), with 10%–40% of patients achieving significant improvement in VA after 1 year of treatment (5, 6). However, a considerable proportion have unsatisfactory response to anti-VEGF agents; 40% of eyes with DME do not or have suboptimal response to anti-VEGF treatment (7, 8). Nonetheless, there is little information to date about the prognostic factors of poor responders.

Optical coherence tomography (OCT) images are readily available to physicians and provide detailed information. Structural changes presumably reflect part of the complex pathophysiologic processes occurring in DME. Furthermore, anatomical measures on spectral-domain (SD) OCT can predict treatment success or failure of various therapies (9). Distinct structural changes identifiable on SD-OCT could reflect part of the intraocular pathophysiologic process change after anti-VEGF treatments and help predict the treatment response.

Among patients with DME refractory to anti-VEGF therapy after a loading dose of three consecutive monthly injections, those who were switched to other treatment modalities (e.g., corticosteroids) had better visual and anatomical outcomes at 12 months than did those who continued with anti-VEGF therapy (10). *Post hoc* analysis from the DRCR.net Protocol I study also indicates that early central macular thickness (CMT) response to anti-VEGF is a significant prognostic indicator of medium to long-term anatomical outcomes in DME (11). Accordingly, the early identification of patients who would not benefit from first-line treatment with anti-VEGF therapy is critical. Real-world studies have become increasingly important in providing evidence of treatment effectiveness in clinical practice. They can therefore provide information on the long-term safety, particularly of rare events, and efficacy of drugs in large heterogeneous populations, as well as information on utilization patterns and health and economic outcomes (12). We aimed to investigate whether the characteristics identified on SD-OCT could be predictive markers of treatment response after three monthly anti-VEGF therapies in DME patients.

## Research design and methods

### Study design and setting

This retrospective study was approved by the Institutional Review Board of the Research Ethics Committee of Hualien Tzu-Chi Hospital and Buddhist Tzu-Chi Medical Foundation (IRB110-188-B) and was conducted in accordance with the guidelines of the Declaration of Helsinki. Data were obtained from Hualien Tzu Chi Hospital Medical Center. Data of patients with DME treated with intravitreal anti-VEGF between April 1, 2013 and April 1, 2021 were reviewed. Written informed consent was obtained from all patients.

### Study participants

The inclusion criteria were as follows (1): age≥ 20 years; (2) type 1 or 2 diabetes mellitus; (3) treatment-naïve DME causing visual loss macular edema defined clinically and as retinal thickness of >300 μm in the central subfield and intraretinal or subretinal fluid seen on SD-OCT; and (4) treatment with anti-VEGF agents. The exclusion criteria were (1) another concomitant ocular disease that causes macular edema (i.e., neovascular age-related macular degeneration or choroidal neovascularization due to other reasons, retinal vein occlusion, uveitis, and recent intraocular surgery possibly causing postsurgical macular edema or influence drug absorption, such as cataract surgery or vitrectomy); (2) previous treatment with intraocular corticosteroids or pan-retinal photocoagulation within 6 months before treatment with anti-VEGF agents. For patients who received bilateral treatment, both the eyes were included. Refractory DME was defined as a reduction of less than 10% in retinal thickness on SD-OCT measured 1 month after at least three monthly anti-VEGF injections. Data on demographic data, age, sex, and type of retinopathy (non-proliferative or proliferative) were collected from patient charts.

### Optical coherence tomography analysis

Qualitative and quantitative evaluations of SD-OCT images encompassing the fovea were performed at baseline and 4 months after treatment to assess the presence of several morphologic features, including (1) SRD (height at the fovea was measured); (2) cystoid changes in the outer nuclear layer (ONL) and maximal cyst size (small <100μm, large 100-200μm, giant >200μm); (3) continuity of the inner segment-outer segment (IS-OS) layer (completely continuous, partly disrupted, completely disrupted); (4) presence of hyperreflective foci (HRF), as well as quantity (few, 2–10; many >11) and location (between the internal limiting membrane and the inner nuclear layer; between the outer plexiform layer and external limiting membrane; in all retinal layers); (5) status of the vitreomacular interface (detached, vitreomacular adhesion [VMA], vitreomacular traction[VMT]); (6) presence of an epiretinal membrane (ERM); (7) CMT; and (8) presence of diffuse retinal thickening (DRT), as well as the width (≦1, 1–3, 3–6 mm). OCT scans were obtained using SD-OCT (Heidelberg Spectralis, Heidelberg, Germany). The listed features were evaluated using a horizontal b-scan encompassing the fovea. The OCT images were evaluated by two experienced retina specialists (MS He and YC Chang) blinded to the outcome. CMT was recorded at baseline and at 1, 2, 3, and 4 months.

### Statistical analysis

Continuous variables and categorical variables were expressed as the mean with standard deviation and as the frequency with proportion, respectively. Both eyes of the patients were included in the analysis. Considering the correlation between eyes, the generalized estimate equation (GEE) was employed for assessing the baseline predictors for the continuous outcome of central macular thickness reduction and central macular thickness reduction <10%. All statistical analyses were performed using SPSS for Windows (version 21.0; IBM, Armonk, NY, USA). All p values were two-sided, and p<0.05 was considered statistically significant.

## Results

### Study participants

A total of 72 eyes from 44 patients were included in the analysis. The demographic patient characteristics are shown in **Table 1**. All eyes with DME had no history of anti-VEGF treatment and were treated with three consecutive monthly intravitreal injections of anti-VEGF. Three main types of anti-VEGF drugs were used in our cohort, the most common of which was ranibizumab (n=60 eyes, 83.3%), followed by aflibercept (n=10, 13.9%) and bevacizumab (n=2, 2.8%). A total of 24 eyes (33.3%) had proliferative diabetic retinopathy (PDR), 43 eyes (59.7%) had severe non-proliferative diabetic retinopathy (NPDR), and 5 eyes (6.9%) had moderate NPDR.

**Table 1:** Descriptive statistics: demographic data and optical coherence tomography baseline measures.

Variables are based on number of subjects		
**Sex**	N (%)	
Female	25 (56.8%)	
Male	19 (43.2%)	
**Age (yrs), Mean (SD)**	62.64 (9.75)	
**HbA1c (%), Mean (SD)**	8.34 (2.09)	
Variables are based on number of eyes		
Baseline Measures	Left eye N=37	Right eye N=35
Diffuse retinal thickening		
3-6mm	15 (40.5%)	17 (48.6%)
1-3mm	17 (45.9%)	6 (17.1%)
≤1mm	3 (8.1%)	1 (2.9%)
0mm (ref.)	2 (5.4%)	11 (31.4%)
ONL cyst size		
Giant	1 (2.7%)	2 (5.7%)
Large	8 (21.6%)	11 (31.4%)
Small	4 (10.8%)	7 (20.0%)
No (ref.)	24 (64.9%)	15 (42.9%)
IS-OS continuity		
Completely disrupted	2 (5.6%)	2 (5.7%)
Partially continuous	13 (36.1%)	16 (45.7%)
Completely continuous	21 (58.3%)	17 (48.6%)
HRF foci-quantity & foci-location		
Many (≥11) & all layers	9 (24.3%)	14 (40.0%)
Many (≥11) & OPL-ELM	1 (2.7%)	0 (0.0%)
Many (≥11) & ILM-INL	1 (2.7%)	3 (8.6%)
Few (2-10) & all layers	8 (21.6%)	5 (14.3%)
Few (2-10) & OPL-ELM	2 (5.4%)	2 (5.7%)
Few (2-10) & ILM-INL	11 (29.7%)	6 (17.1%)
Absent	5 (13.5%)	5 (14.3%)
Vitreomacular interface		
VMT	1 (2.8%)	0 (0.0%)
VMA	30 (83.3%)	34 (90.1%)
PVD	5 (13.9%)	1 (2.9%)
ERM		
Yes	7 (18.9%)	8 (22.9%)
No (ref.)	30 (81.1%)	27 (77.1%)
SRD		
Yes	5 (13.5%)	11 (31.4%)
No	32 (86.5%)	24 (68.6%)
DR type		
PDR	13 (35.1%)	11 (31.4%)
Severe NPDR	22 (59.5%)	21 (60.0%)
Moderate NPDR	2 (5.4%)	3 (8.6%)

ELM, external limiting membrane; ERM, epiretinal membrane; ref, reference; HRF, hyperreflective foci; ILM, internal limiting membrane; IS-OS, inner segment-outer segment; NPDR, non- proliferative diabetic retinopathy; ONL, outer nuclear layer; OPL, outer plexiform layer; PDR, proliferative diabetic retinopathy; PVD, posterior vitreous detachment; SD, standard deviation; SRD, serous retinal detachment; VMA, vitreomacular adhesion; VMT, vitreomacular traction.

### Anatomic baseline characteristics

The baseline OCT characteristics are shown in **Table 1**. With respect to the DME morphology, DRT was the most common presentation (n=59 eyes, 81.9%), followed by cystoid macular edema (CME) (n=33 eyes, 45.8%) and SRD (n=16 eyes, 22.2%). Furthermore, eyes with DME were more commonly to present with complete continuous IS-OS continuity (52.8%), HRF (86.1%), and VMA (88.9%) in the baseline.

### Optical coherence tomography predictors for treatment response

**Figure 1:**
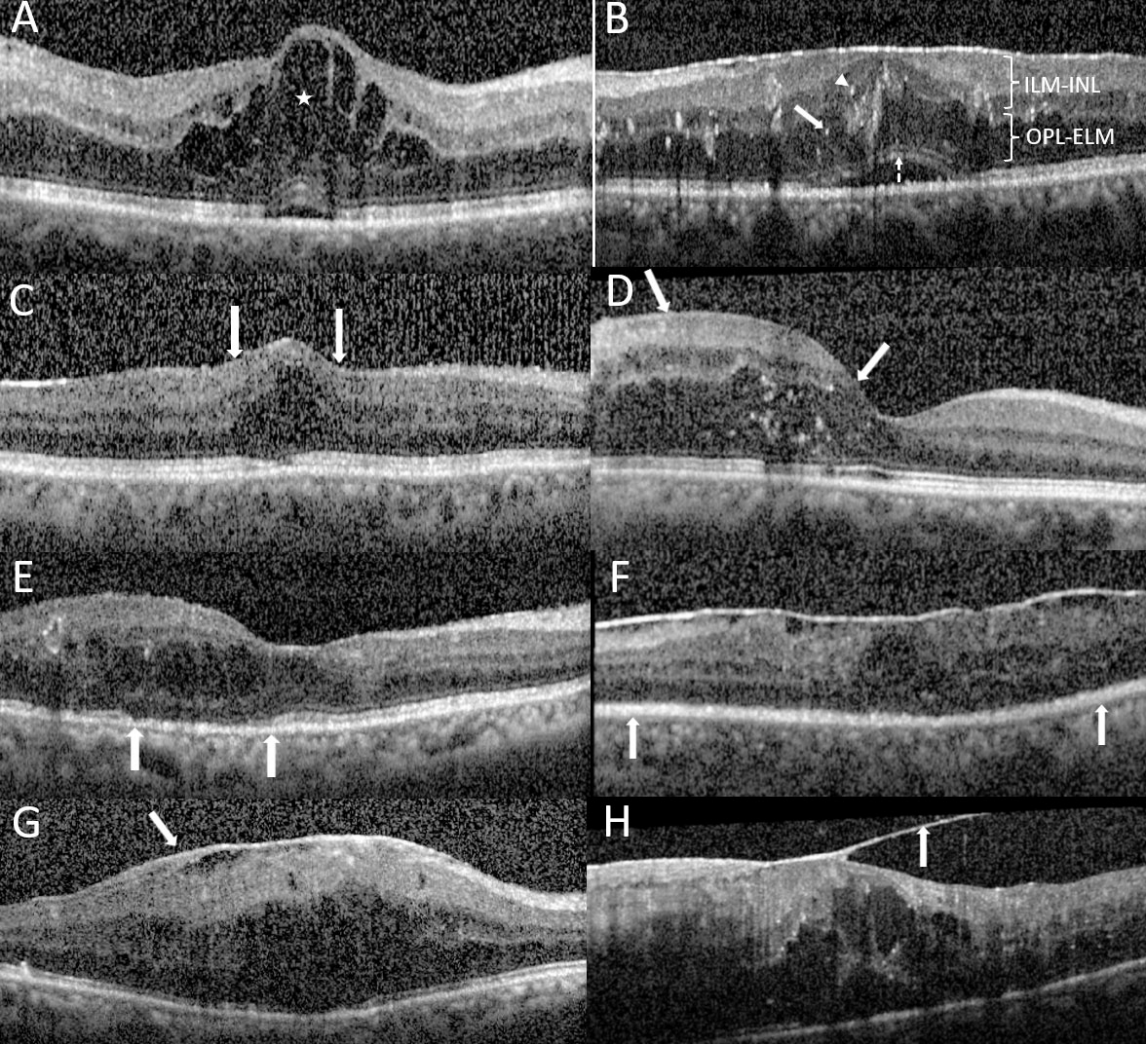
OCT measures. **(A)**, Grading of outer nuclear layer (ONL) cysts: Cystoid diabetic macular edema (DME) with a giant ONL cyst (★). **(B)**, Serous retinal detachment (SRD) with diffuse retinal thickening (DRT, width of 3–6mm) showing retinal elevation between the sensory retina and the retinal pigment epithelium (dashed arrow); the height of SRD is measured. Grading of hyperreflective foci (HRF): A high number of HRF (≥11) are distributed in all layers (located between the ILM and INL [arrowhead] and between OPL and ELM [arrow]). **(C, D)**, Grading of DRT: **(C)**, DME associated with focal DRT (width ≦1 mm, between arrows). **(D)**, DME related to localized DRT (width within 1–3 mm, between arrows). **(E, F)**, Grading of the inner segment-outer segment (IS-OS) integrity. **(E)**, Partially disrupted continuity of the IS-OS layer (between arrows). **(F)**, Complete discontinuity of the IS-OS layer (between arrows). **(G)**, DME associated with epiretinal membrane (arrow). **(H)**, DME associated with vitreomacular traction (arrow). ELM, external limiting membrane; ILM, internal limiting membrane; INL, inner nuclear layer; OPL, outer plexiform layer.

**Figure 2:**
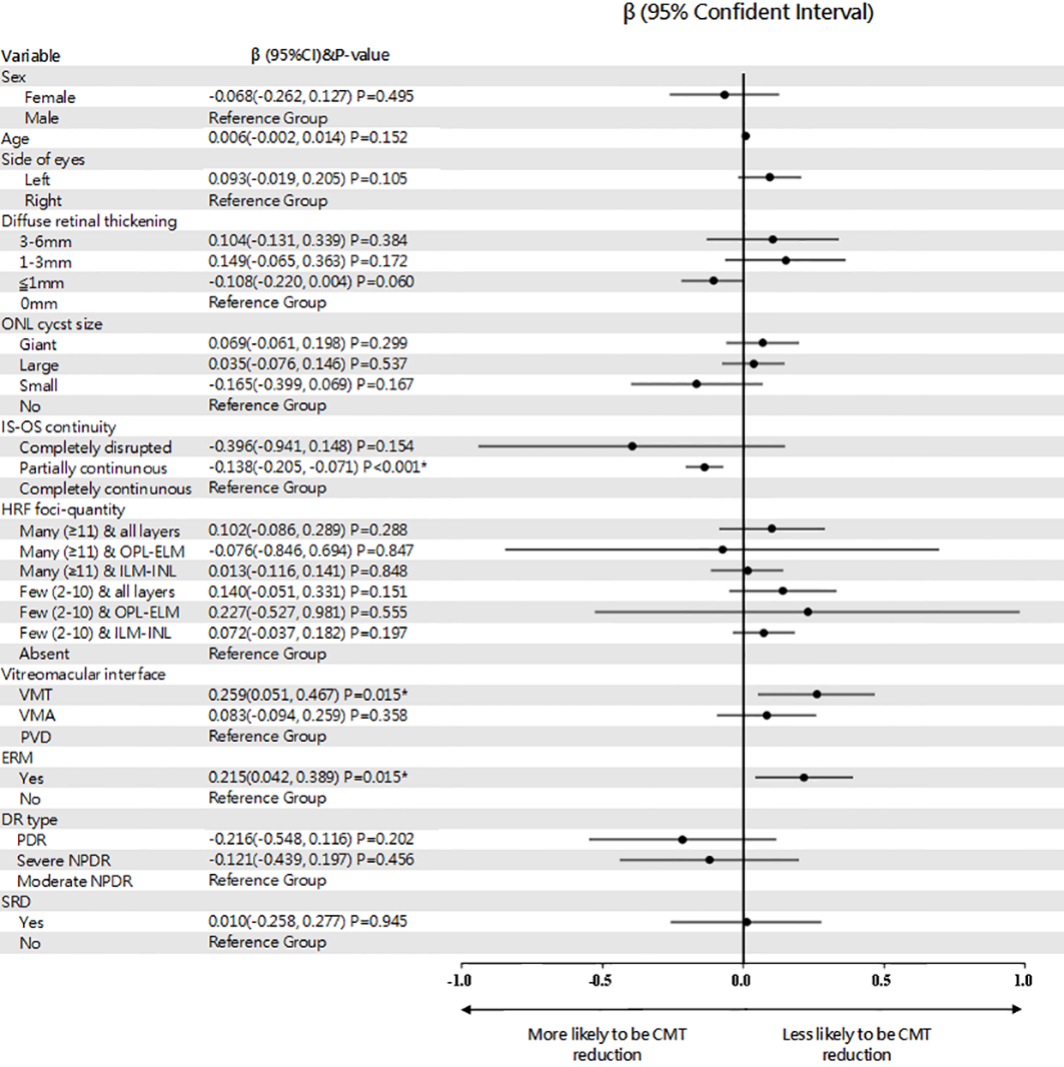
Forest plot of baseline predictors of CMT reduction, using generalized estimate equation model. CMT, central macular thickness; ONL, outer nuclear layer; IS-OS, inner segment-outer segment; HRF, hyperreflective foci; OPL, outer plexiform layer; ELM, external limiting membrane; ILM, internal limiting membrane; VMA, vitreomacular adhesion; VMT, vitreomacular traction; PVD, posterior vitreous detachment; ERM, epiretinal membrane; PDR, proliferative diabetic retinopathy; NPDR, non-proliferative diabetic retinopathy; SRD, serous retinal detachment; β, beta coefficient. * Statistically significant at p<0.05.

**Figure 3:**
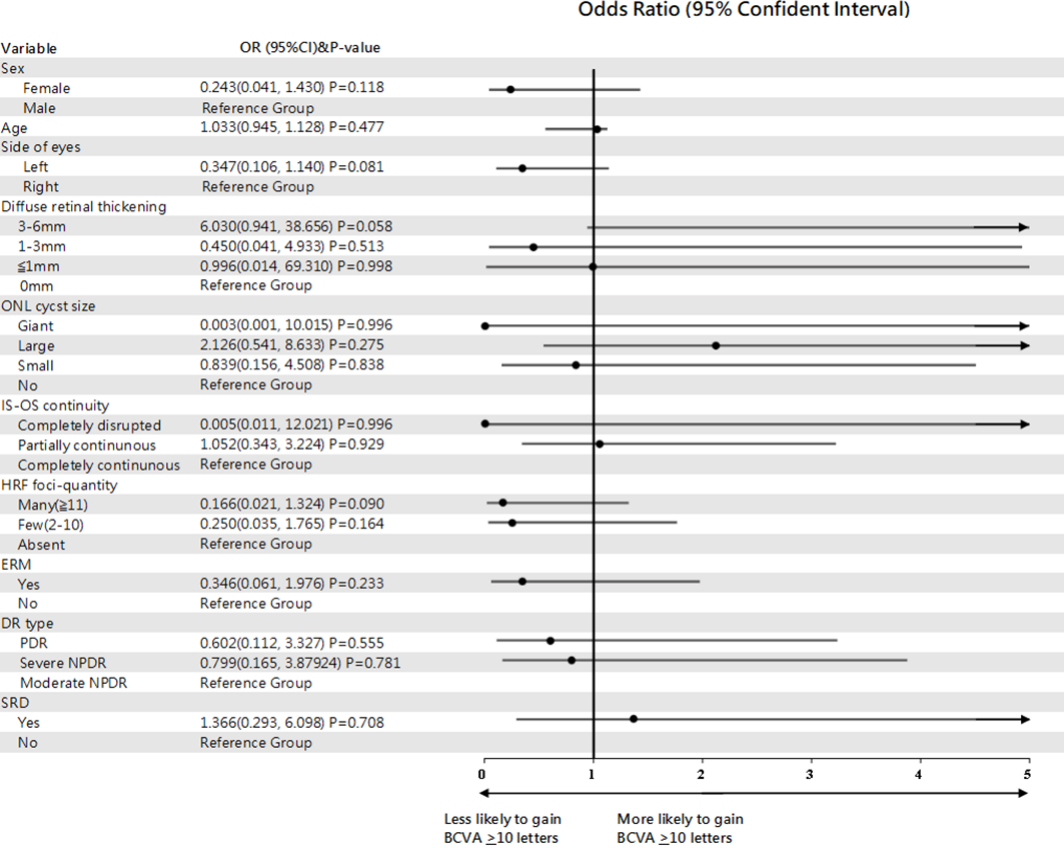
Forest plot of baseline predictors of a ≥10 letter gain in BCVA, using generalized estimate equation model. BCVA, best-corrected visual acuity; ONL, outer nuclear layer; IS-OS, inner segment-outer segment; HRF, hyperreflective foci; ERM, epiretinal membrane; SRD, serous retinal detachment.

Eyes with partially continuous IS-OS layers had a better treatment response after 4 months (partially vs. completely continuous: β=-0.138; Wald chi-square=16.392; P<0.001). Baseline VMT was a predictor of poor functional treatment response after 4 months (VMT vs. posterior vitreous detachment: β=0.259; Wald chi-square=5.938; P=0.015). Moreover, eyes with ERM at baseline were more likely to have poor response at 4 months (present vs. absent ERM: β=0.215; Wald chi-square=5.921; P=0.015). **Figure 1** shows the OCT biomarkers that were predictive of treatment response after 4 months. The predictive values of all OCT measures examined are shown in **Table 2**. Baseline predictors of mean CMT reduction are shown in **Figure 2**. Furthermore, the odds of gaining BCVA ≥10 letters at 4 months trended toward the OCT predictive value of CMT reduction; however, this was not significant (**Table 3**). All OCT biomarkers that were predictive of good BCVA response are shown in **Figure 3**.

**Table 2:** Baseline predictors for the mean reduction (improvement) of central macular thickness.

Variable	β	Wald Chi-Square	P-value
Sex			
Female	-0.068	0.465	0.495
Male (ref.)	−		
**Age**	0.006	2.053	0.152
Diffuse retinal thickening			
3-6mm	0.104	0.756	0.384
1-3mm	0.149	1.864	0.172
≤1mm	-0.108	3.542	0.060
0mm (ref.)	−		
ONL cyst size			
Giant	0.069	1.080	0.299
Large	0.035	0.381	0.537
Small	-0.165	1.908	0.167
No (ref.)	−		
**IS-OS continuity**			
Completely disrupted	-0.396	2.034	0.154
Partially continuous	-0.138	16.392	<0.001
Completely continuous (ref.)	−		
HRF foci-quantity			
Many (≥11) & all layers	0.102	1.130	0.288
Many (≥11) & OPL-ELM	-0.076	0.037	0.847
Many (≥11) & ILM-INL	0.013	0.037	0.848
Few (2-10) & all layers	0.140	2.062	0.151
Few (2-10) & OPL-ELM	0.227	0.348	0.555
Few (2-10) & ILM-INL	0.072	1.666	0.197
Absent (ref.)	−		
Vitreomacular interface			
VMT	0.259	5.938	0.015
VMA	0.083	0.845	0.358
PVD (ref.)	−		
ERM			
Yes	0.215	5.921	0.015
No (ref.)	−		
DR type			
PDR	-0.216	1.631	0.202
Severe NPDR	-0.121	0.555	0.456
Moderate NPDR (ref.)	−		
SRD			
Yes	0.010	0.005	0.945
No (ref.)	−		

ELM, external limiting membrane; ERM, epiretinal membrane; ref, reference; HRF, hyperreflective foci; ILM, internal limiting membrane; IS-OS, inner segment-outer segment; NPDR, non- proliferative diabetic retinopathy; ONL, outer nuclear layer; OPL, outer plexiform layer; PDR, proliferative diabetic retinopathy; PVD, posterior vitreous detachment; SRD, serous retinal detachment; VMA, vitreomacular adhesion; VMT, vitreomacular traction; β, beta coefficient.

**Table 3:** Baseline predictors of a ≥10 letter gain in best-corrected visual acuity.

Variable	OR	95% CI		P-value
		Upper	Lower	
Sex				
Female	0.243	0.041	1.430	0.118
Male (ref.)	1	.	.	
**Age**	1.033	0.945	1.128	0.477
Diffuse retinal thickening				
3-6mm	6.030	0.941	38.656	0.058
1-3mm	0.450	0.041	4.933	0.513
≤1mm	0.996	0.014	69.310	0.998
0mm (ref.)	1			
ONL cyst size				
Giant	0.003	0.001	10.015	0.996
Large	2.126	0.541	8.633	0.275
Small	0.839	0.156	4.508	0.938
No (ref.)	1			
IS-OS continuity				
Completely disrupted	0.005	0.011	12.021	0.996
Partially continuous	1.052	0.343	3.224	0.929
Completely continuous (ref.)	1			
HRF foci-quantity				
Many (≥11)	0.166	0.021	1.324	0.090
Few (2-10)	0.250	0.035	1.765	0.164
Absent (ref.)	1			
ERM				
Yes	0.346	0.061	1.976	0.233
No (ref.)	1			
DR type				
PDR	0.602	0.112	3.237	0.555
Severe NPDR	0.799	0.165	3.879	0.781
Moderate NPDR (ref.)	1			
SRD				
Yes	1.366	0.293	6.098	0.708
No (ref.)	1			

CI, confidence interval; ERM, epiretinal membrane; ref, reference; HRF, hyperreflective foci; IS-OS, inner segment-outer segment; NPDR, non- proliferative diabetic retinopathy; ONL, outer nuclear layer; OR,Odds ratio; PDR, proliferative diabetic retinopathy; PVD, posterior vitreous detachment; SRD, serous retinal detachment; VMA, vitreomacular adhesion; VMT, vitreomacular traction.

**Figure 4:**
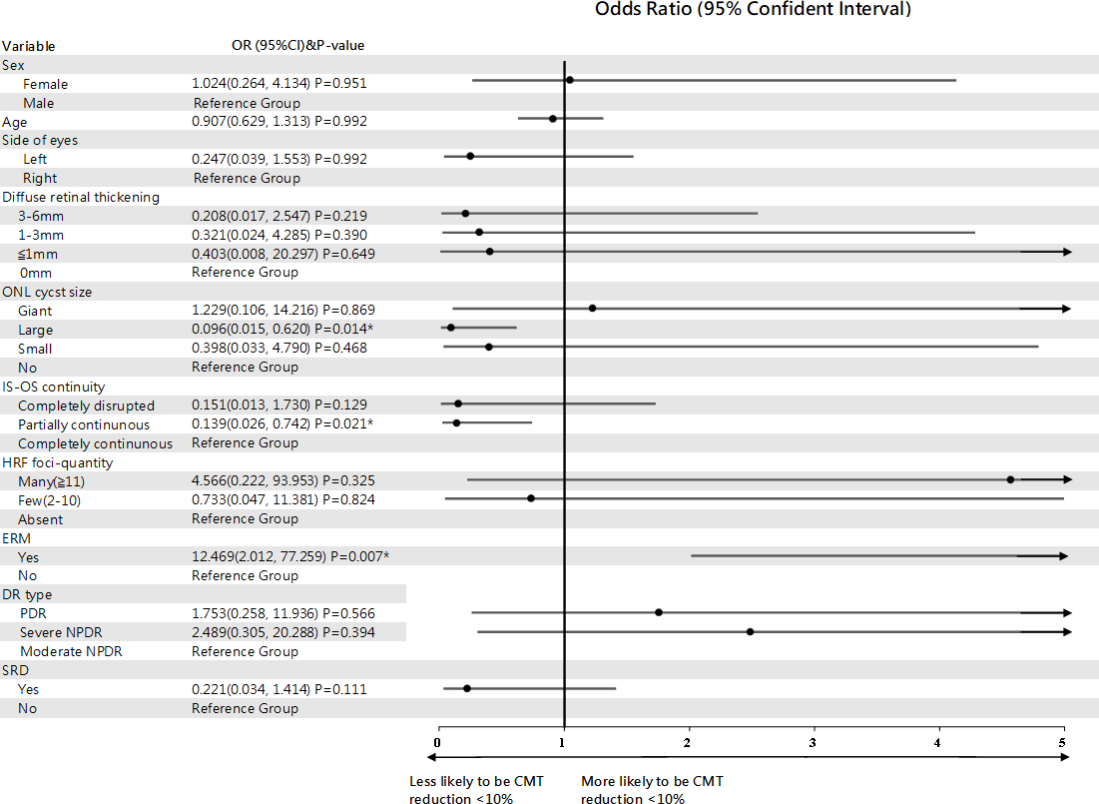
Forest plot of baseline predictors of CMT reduction <10%, using generalized estimate equation model. CMT, central macular thickness; ONL, outer nuclear layer; IS-OS, inner segment-outer segment; HRF, hyperreflective foci; ERM, epiretinal membrane; SRD, serous retinal detachment. *Statistically significant at p<0.05.

In a subanalysis, eyes with CMT reduction of less than 10% after 4 months were designated to the refractory group (**Table 4**). The results showed that ERM at baseline predicted highly increased odds of poor response at 4 months (OR, 12.469; 95% CI, 2.012–77.259; P=0.007). In contrast, large ONL cyst sizes at baseline (OR, 0.096; 95% CI, 0.015–0.62; P=0.014) and partially continuous IS-OS layers (OR, 0.139; 95% CI, 0.026–0.742; P=0.021) were less likely to be refractory group after 4 months (**Figure 4**).

**Table 4:** Baseline predictors of central macular thickness reduction <10%.

Variable	OR	95% CI		P-value
		Upper	Lower	
Sex				
Female	1.044	0.264	4.134	0.951
Male (ref.)	1			
**Age**	0.907	0.629	1.313	0.411
Diffuse retinal thickening				
3-6mm	0.208	0.017	2.547	0.219
1-3mm	0.321	0.024	4.285	0.390
≤1mm	0.403	0.008	20.297	0.649
0mm (ref.)	1			
ONL cyst size				
Giant	1.229	0.106	14.216	0.869
Large	0.096	0.015	0.620	0.014
Small	0.398	0.033	4.790	0.468
No (ref.)	1			
IS-OS continuity				
Completely disrupted	0.151	0.013	1.730	0.129
Partially continuous	0.139	0.026	0.742	0.021
Completely continuous (ref.)	1			
HRF foci-quantity				
Many (≥11)	4.566	0.222	93.953	0.325
Few (2-10)	0.733	0.047	11.381	0.824
Absent (ref.)	1			
ERM				
Yes	12.469	2.012	77.259	0.007
No (ref.)	1			
DR type				
PDR	1.753	0.258	11.936	0.566
Severe NPDR	2.489	0.305	20.288	0.394
Moderate NPDR (ref.)	1			
SRD				
Yes	0.221	0.034	1.414	0.111
No (ref.)	1			

CI, confidence interval; ERM, epiretinal membrane; ref, reference; HRF, hyperreflective foci; IS-OS, inner segment-outer segment; NPDR, non- proliferative diabetic retinopathy; ONL, outer nuclear layer; OR, Odds ratio; PDR, proliferative diabetic retinopathy; PVD, posterior vitreous detachment; SRD, serous retinal detachment; VMA, vitreomacular adhesion; VMT, vitreomacular traction.

In the univariate analysis, SRD at baseline was significantly associated with treatment response to anti-VEGF agents (MD, -188.69 μm; 95% CI, -128.95 to -248.43; P<0.001) (**Table 5**). However, in multivariate survival analysis, treatment response to anti-VEGF regimens was not a significant influencing factor in patients with SRD (present vs. absent: β, 0.01; Wald chi-square, 0.005; P=0.945) (**Table 2**).

**Table 5:** Analysis for mean change of subfoveal serous retinal detachment (μm) at 4 months.

	N	Mean	SD	MD	95%CI	P-value
SRD (baseline)	32	207.06	139.37	-188.69	-248.43, -128.95	<0.001
SRD (at 4 months)		18.38	51.90			

CI, confidence interval; MD, mean difference; SD, standard deviation; SRD, serous retinal detachment.

## Discussion

In this real-world evidence-based study, we identified partially continuous IS-OS layers as biomarkers that predict better response to anti-VEGF therapy in DME. In contrast, ERM is a significant predictor of poor response. DME has a complex pathogenesis, with multiple factors contributing to its pathophysiology, including angiogenic, inflammatory, hypoxic, and hemodynamic processes that lead to the breakdown of the blood-retinal barrier (BRB) and leakage of intraretinal fluid (13). Anti-VEGF injections are generally recommended as the first-line therapy for DME; however, refractory cases are not uncommon. A *post-hoc* analysis of the DRCR.net Protocol I reported an approximately 40% prevalence of refractory DME after 2 years of monthly intravitreal ranibizumab treatment (7). Combined data from the RIDE/RISE trial found that 23% of eyes receiving intravitreal ranibizumab had persistent macular edema at the end of the study period (14). The real-world prevalence of refractory DME may be higher than estimated in these studies, as rigorous enrolment and follow-up protocols in clinical trials are unlikely to be fully replicated in everyday practice (15). An important issue is the possibility of early identification of patients who would not benefit from first-line anti-VEGF therapy.

VEGF is significantly higher in all types of DME than that in the eyes of non-diabetes patients, indicating that VEGF is equally important for any morphological changes in eyes with DME (16). However, evidence indicates that bioactive factors such as cytokines are also released into the retina. The proposed pathophysiology of each type is quite different; thus, each DME type has its own morphological and topographic characteristics (17). interleukin (IL)-6, a pro-inflammatory cytokine, intraocular levels of IL-6 were significantly higher in eyes with SRD than in eyes with DRT or CME, implying active inflammation. A recent study showed a better response to dexamethasone implants in eyes with SRD (9). The predictive value of SRD at baseline for the treatment response to anti-VEGF agents in DME remains controversial. Although some studies reported a significant improvement in VA in patients with SRD at baseline (18, 19), others found no difference or even worse functional results (20, 21). Univariate analysis to assess whether SRD is responsive to anti-VEGF agents in the current study showed that SRD significantly responded to anti-VEGF agents. However, in the multivariate survival analysis, treatment response to anti-VEGF regimens was not a significant influencing factor in patients with SRD. This can happen when SRD and other covariates are highly correlated. Furthermore, additional variables (ex. ERM) may explain more of the variance in the outcome variable, and thus reduce the impact of the initially significant of SRD.

In DME, the concentration of intraocular VEGF is significantly correlated with IL-6 levels (16). Anti-VEGF therapy reduces intraocular subclinical inflammation, and the aqueous humor concentration of IL-6 is decreased after anti-VEGF treatment (22). This could explain the response to anti-VEGF therapy in eyes with SRD in the current study. Our result was also consistent with that in the study by Sophie et al. (18) who reported that suppression of VEGF effectively eliminated subretinal fluid. Future prospective comparative investigations of the efficacy of anti-VEGF and of dexamethasone implants in eyes with SRD are required to optimize patient management.

In the current study, 45.8% of all patients presented with ONL cysts, and the majority had large ONL cysts (100–200 µm), mainly occurring at a relatively late stage of the disease. We found that large ONL cysts at baseline are less likely to be refractory group after anti-VEGF treatment at 4 months. Previous studies have reported that large ONL cysts negatively affect macular function and are predictive of worse VA outcomes after anti-VEGF therapy (23, 24). Elevated VEGF levels in DR affect the inner BRB, leading to increased vascular permeability, decreased osmotic gradient, extracellular fluid accumulation, and cyst formation (25). Furthermore, liquefaction necrosis of Müller cells and related inflammatory factors result in fluid accumulation in the cystic space (17). However, unlike SRD, IL-6 and IL-8 levels were not significantly increased in eyes with cystic changes (16). This indicated that the eye may not be in an active inflammatory state; rather, it could be a remnant of a previous inflammatory reaction (16). Anti-VEGF agents have been shown to decrease permeability and improve inner BRB by interacting with junctional proteins in the vascular endothelium (23). Our results support this finding and are consistent with a recent report that throughout anti-VEGF treatment, significant regression of ONL cysts accompanied notable improvement of macular function with a substantial decrease in their size (23). Nevertheless, we could not find an association between large ONL cysts and a mean reduction of CMT after anti-VEGF treatment at four months. Furthermore, only 3 eyes presented with giant ONL cysts (>200 µm), and we were unable to find an association between treatment outcomes and anti-VEGF agents in patients with giant ONL cysts.

The pathogenesis of DRT involves the persistent breakdown of the inner BRB and impairment of fluid absorption by Müller cells (17). DRT can be localized or more diffusely encompass the macula. Previous studies have reported that intravitreal anti-VEGF therapy is more effective for the DRT type than for the other types of DME (20, 26). Nevertheless, no study assessed whether the degree of the DRT would interfere with the treatment response or not. To clarify the relationship between DRT and response to anti-VEGF treatment, we first qualitatively evaluated the width of the DRT on a standard horizontal 6-mm B-scan OCT centered through the fovea and further stratified it into three subgroups (≤ 1, 1–3, and 3–6 mm). The results showed that the width of DRT was inversely proportional to the odds of poor response. Specifically, there was a trend indicating that the degree of DRT was proportionally associated with better response, although this was not significant in multivariate analysis.

There is a high incidence of vitreomacular interface abnormality (VMIA) among DME patients (27). The current study found a 20.8% incidence of ERM in our cohort. Although DR and its severity are risk factors for developing secondary ERM (27, 28), cases of VMIA are excluded from major clinical trials, even though DME is associated with this condition in 25% of patients (29). Nevertheless, knowledge regarding the effect of VMIA on the response to anti-VEGF treatment in patients with DME has not been thoroughly investigated. Ercalik et al. retrospectively evaluated 56 eyes with or without ERM and found a negative effect of ERM on intravitreal anti-VEGF treatment (30). Wong et al. conducted a prospective study of 104 eyes with DME treated with anti-VEGF and found that ERM was associated with a worsened visual and anatomic response (31). Notably, neither study considered other OCT biomarkers; thus, the findings might not completely represent the true impact of ERM in eyes with DME.

Considering the diversity of OCT morphology in DME, we included various OCT biomarker characteristics and considered ERM as a variable in eyes with refractory DME, despite anti-VEGF therapy. Furthermore, we used a multivariate statistical model to analyze the association between treatment response and each OCT biomarker. Our results showed that ERM significantly increased the odds of poor response. According to previous study on ERM pathology in diffuse DME, multilayered membranes are mainly composed of hyalocytes and myofibroblasts. Hyalocytes were shown to produce VEGF and can transdifferentiate into myofibroblasts, known for their contractive properties (32). Furthermore, contraction of the ERM may cause perifoveal capillary leakage and aggravate macular edema. It has also been demonstrated that glial cells in ERM produce various cytokines and growth factors. VEGF and its receptors, as well as IL-6, are localized to cells in the ERM of patients with DR, thus further increasing inflammation and possibly promoting DME persistence (30, 33). Furthermore, ERM may act as a physical barrier and decrease drug penetration after intravitreal injections of anti-VEGF in DME treatment (34).

The connective tissue growth factor (CTGF) is one of the most potent profibrotic factors. It can stimulate fibroblast proliferation and collagen deposition, resulting in fibrosis (35). Anti-VEGF has been reported to cause hypoxia in vascular endothelial cells and increase CTGF expression, which plays an important role in ERM formation (36). As a result, anti-VEGF therapy may potentially aggravate ERM contractions and interfere with the resolution of macular edema in diabetes. These results may explain the increased likelihood of poor response in this group. Nevertheless, consistent with the guidelines for DME management by retinal specialists, PPV is currently recommended as a therapeutic option in cases of DME associated with VMT (37). In the absence of traction formation, there is no consensus on the role of PPV in the actual treatment of diabetic eyes. Our results call for further comparative studies and treatment modalities other than anti-VEGF in DME patients presenting with ERM-impaired visual and anatomic outcomes.

HRF represents subclinical lipoproteins that extravasate after inner BRB breakdown. It is initially present in the inner retinal layers and subsequently migrates to the outer retinal layers (38). HRF is an important imaging marker for retinal inflammation (39). However, the predictive value of HRF for visual outcomes after anti-VEGF treatment in DME is unclear (9). In our study, we did not find that the presence of HRF was associated with treatment response after anti-VEGF therapy. The integrity of outer retinal layers is a direct indicator of the health of the retinal photoreceptors and retinal pigment epithelium. IS-OS integrities is an important factor for predicting VA after treatment. Subjects with long-standing DME may demonstrate focal or diffuse loss of integrity of the IS-OS junction. Previous studies have reported that IS-OS integrity can be expected to recover after anti-VEGF therapy (17, 23). Our results support this finding and show a better response to anti-VEGF therapy in eyes with partially continuous IS-OS layers.

Our study has some limitations. First, its retrospective and non-randomized design precluded a well-matched control enrollment. Second, the sample size was small, which may have hindered the significance of the results. Third, we prescribed three anti-VEGF agents to treat DME in the real-world clinical practice setting. Although most eyes were treated with ranizucimumab (83.3%), we did not assess each anti-VEGF regimen separately, and thus, the different efficacy between each agent may not have been accounted. Fourth, in our study, despite the odds of gaining BCVA ≥10 letters trended toward the OCT predictive value of CMT reduction, no OCT biomarkers showed significant predictive value for good BCVA response at 4 months. We found that the OCT predictive value of CMT reduction cannot fully translate into the change of VA, which was consistent with the study of a *post hoc* analysis of the protocol T randomized clinical trial (40). They found changes in CMT appear to account for only a small proportion of the total variation in changes in BCVA, and concluded that changes in CMT cannot support as a surrogate for changes in VA in evaluating anti-VEGF for DME. Despite these limitations, an important strength of our study is that we included various common OCT markers in patients with DME, which yielded ample information and helped us tailor timely and individualized treatment during daily practice.

In conclusion, partial IS-OS continuity is the marker that predicts better response to anti-VEGF treatment in eyes with DME. In contrast, the presence of ERM is a significant predictor of poor response. Our results raise the pertinent issue that DME patients with ERM are significant poor responders to anti-VEGF therapy and may benefit more from other therapeutic approaches.

## Data availability statement

The original contributions presented in the study are included in the article/supplementary material. Further inquiries can be directed to the corresponding author.

## Ethics statement

The studies involving human participants were reviewed and approved by Institutional Review Board of the Research Ethics Committee of Hualien Tzu-Chi Hospital and Buddhist Tzu-Chi Medical Foundation. The patients/participants provided their written informed consent to participate in this study.

## Author contributions

T-CH and M-SH had full access to all of the data in the study and take responsibility for the integrity of the data and the accuracy of the data analysis. Concept and design: M-SH. Acquisition, analysis, or interpretation of data: T-CH, G-HD, Y-CC, and M-SH. Drafting of the manuscript: T-CH, G-HD, and M-SH. Critical revision of the manuscript for important intellectual content: T-CH, F-LC, and M-SH. Statistical analysis: T-CH and G-HD. Administrative, technical, or material support: G-HD, M-SH, and Y-CC. Supervision: T-CH, M-SH. All authors contributed to the article and approved the submitted version.
